# Comparative Analysis of Genetic Parameters for Test-Day Egg Production in Four Thai Native Synthetic Chicken Lines Under Heat Stress

**DOI:** 10.3390/ani15192912

**Published:** 2025-10-07

**Authors:** Doungnapa Promket, Khanitta Pengmeesri, Vibuntita Chankitisakul, Wuttigrai Boonkum

**Affiliations:** 1 Branch of Animal Science, Department of Agricultural Technology, Faculty of Technology, Mahasarakham University, Mahasarakham 44150, Thailand; napakran@hotmail.com (D.P.); khanitta.c@msu.ac.th (K.P.); 2 Applied Animal and Aquatic Sciences Research Unit, Faculty of Technology, Mahasarakham University, Mahasarakham 44150, Thailand; 3 Department of Animal Science, Faculty of Agriculture, Khon Kean University, Khon Kean 40002, Thailand; vibuch@kku.ac.th; 4 Network Center for Animal Breeding and Omics Research, Khon Kaen University, Khon Kaen 40002, Thailand

**Keywords:** egg production, genetic improvement, genetic parameters, synthetic chicken lines, heat tolerance, heat stress

## Abstract

**Simple Summary:**

Heat stress poses a significant challenge to egg production in tropical climates. This study evaluated four Thai native synthetic chicken lines for their ability to maintain egg laying under increasing temperature and humidity. While all lines showed reduced egg production under heat stress, the magnitude of decline varied: Soi Nin was the most resilient, whereas Kaimook e-san was the most affected. These findings highlight the potential of selecting and breeding heat-tolerant lines to sustain egg production, supporting both food security and farmers’ income in hot regions.

**Abstract:**

This study evaluated genetic parameters for test-day egg production in four Thai native synthetic chicken lines—Soi Nin, Soi Pet, Kaen Thong, and Kaimook e-san—under heat stress in Thailand. A total of 11,887 monthly test-day egg records from 1134 hens, collected between January 2023 and July 2025, were analyzed using a repeatability test-day model with the temperature–humidity index (THI) as an environmental covariate. THI thresholds from 70 to 80 were evaluated, and the THI1 equation provided the best model fit with the highest coefficient of determination (R^2^) and the lowest mean squared error (MSE). With increasing THI, heritability estimates declined from 0.255–0.323 at THI 70 to 0.173–0.236 at THI 80, a 26.9–32.2% decrease reflecting reduced additive genetic variance and consequent lower genetic expression under heat stress. Genetic correlations between egg production and heat stress were positive at low THI (0.250–0.600) but became negative at THI ≥ 73, suggesting antagonism between productivity and thermotolerance under severe stress. The rate of decline in egg production increased with increasing THI, from −0.35 to −0.45 eggs/bird/THI at THI 73, −0.80 to −1.22 at THI 76, and −1.76 to −2.35 at THI 80. The ranges of heritability and decline rates reflect the variation observed among the four Thai native synthetic chicken lines examined in this study. Kaimook e-san consistently showed the steepest decline in egg production, whereas Soi Nin exhibited the smallest, indicating greater resilience. These findings reveal significant genetic variation in heat tolerance among Thai native synthetic lines and underscore the need to consider both productivity and environmental sensitivity in breeding programs to sustain egg production under future climate change.

## 1. Introduction

Egg production is a vital economic trait in chickens, contributing significantly to food security, rural livelihoods, and the global poultry industry [1,2]. In Thailand, native chicken breeds and their synthetic lines are increasingly valued for their adaptability, disease resistance, and meat and egg quality preferred by local consumers [3,4,5,6]. In particular, synthetic lines (Soi Nin, Soi Pet, Kaen Thong, and Kaimook e-san), developed through crossbreeding indigenous and commercial strains, provide a valuable genetic resource for sustainable production in tropical environments [7,8,9]. Each line was established through controlled crossbreeding between local native chicken ecotypes (specifically the Thai native Shee breed) and commercial broilers (comprising 50% Thai native and 50% commercial broiler genetics), followed by within-line selection over seven generations. The breeding program aimed to improve specific traits related to production performance, including growth rate, egg production, and heat tolerance. However, heat stress remains a major constraint on poultry productivity in tropical and subtropical regions [10,11,12]. Global warming and rising ambient temperatures have increased the frequency and severity of heat stress events, which reduce feed intake, alter metabolic processes, and impair reproductive physiology, ultimately lowering egg production and disrupting laying patterns [13,14]. During the laying season, temperatures often exceed 34 °C—well above the poultry thermo-neutral zone of 18–24 °C [14,15]—posing serious challenges to both productivity and animal welfare [16].

While commercial breeds experience sharp declines in productivity under thermal stress, Thai native chickens and their synthetic lines exhibit some degree of natural tolerance [17,18]. However, performance under heat stress still varies both within and between lines, highlighting the need to quantify genetic variability and identify superior genotypes for heat tolerance [19,20]. Several studies have examined the effects of heat stress on poultry performance and genetic parameters. Loyau et al. [21] and Loengbudnark et al. [22] demonstrated that high temperatures reduce heritability estimates for egg traits in laying hens, indicating strong modulation of environmental influence on genetic expression. Moreover, studies in tropical countries, such as Nigeria [23], Jamaica [24], and Egypt [25], have incorporated the temperature–humidity index (THI) into animal models to quantify the impact of heat stress. Additionally, some genomic studies have identified genetic markers associated with heat tolerance in native chickens [18,26,27].

The utility of test-day models in layer chickens has been well demonstrated, showing improved genetic accuracy and model fit when evaluating monthly egg records in commercial breeder hens [28,29]. However, data remain limited for native and synthetic lines under fluctuating environmental conditions. Integrating test-day egg production with environmental covariates, such as THI, offers an important advancement for poultry breeding under heat stress. In the context of climate change, genetic improvement, and food production, understanding the genetic architecture of test-day egg production under heat stress is both timely and essential. This knowledge enables breeders to identify heat-tolerant individuals that maintain stable egg yields, thereby supporting productivity and sustainability. Therefore, this study aimed to estimate genetic parameters for monthly egg production in four Thai native synthetic chicken lines under natural heat stress using THI and to compare their genetic performance and environmental sensitivity with respect to laying consistency, heat resilience, and selection potential in tropical conditions.

## 2. Materials and Methods

This study was reviewed and approved by the Institutional Animal Care and Use Committee, Khon Kaen University, in accordance with the Animal Experimentation Guidelines of the National Research Council of Thailand (Approval No. IACUC-KKU-103/65; 27 December 2022). The study was conducted at the experimental farm of the Network Center for Animal Breeding and Omics Research, Faculty of Agriculture, Khon Kaen University, Thailand.

### 2.1. Data Collection and Data Management

The dataset comprised 11,887 monthly test-day egg production records collected from 1134 individual birds, which were derived from four Thai native synthetic lines (233 Soi Nin, 354 Soi Pet, 181 Kaen Thong, and 366 Kaimook e-san) maintained at the Network Center for Animal Breeding and Omics Research (NCAB), Faculty of Agriculture, Khon Kaen University. The experiment was conducted from January 2023 to July 2025. The four lines have been previously described by Promwatee et al. [30]. At hatch, all chicks were leg-tagged for initial identification, brooded for 4 weeks, and then fitted with wing tags for permanent identification. Chickens were vaccinated against infectious bronchitis, Newcastle disease, fowl pox, and fowl cholera following the Network Center for Animal Breeding and Omics Research (NCAB) vaccination program. They were reared in an open-housing system with an average of 12 h of natural light per day. During brooding (0–4 weeks), chickens received a commercial starter diet [19% crude protein, 2900 kcal metabolizable energy (kcal ME/kg)] followed by a grower diet (15% crude protein, 2900 kcal ME/kg) from 4 to 20 weeks of age, with *ad libitum* access to fresh water. At 20 weeks, hens were transferred to battery cages (20 cm × 45 cm × 40 cm), in compliance with the animal welfare guidelines established by the Department of Livestock Development, Thailand, and egg production was recorded from the first egg (approximately 180 days of age) until 365 days of laying. During the laying period, hens were fed 110 g/d of feed (17% crude protein, 2750 kcal ME/kg) with free access to fresh water.

Before statistical analysis, the raw data from the experimental farm were validated. Normality was assessed using the Shapiro–Wilk test and homogeneity of variance using Levene’s test, followed by outlier removal. Meteorological data, recorded every 3 h at the Khon Kaen Meteorological Station (approximately 3 km from the NCAB chicken farm), were obtained from the Thai Meteorological Department. These data were used to calculate the THI using the following four equations: THI1: $\left(1.8\;\times \;T_{avg}+32\right)-\left(0.55-0.0055\;\times \;RH_{Avg}\right)\times \left(1.8\times T_{avg}-26\right)$ [31], THI2: 0.85(DB)+ 0.15(WB) [32], THI3: $0.60(T_{max\;})+\;0.40(T_{min\;})$ [33], THI4: $DB-[\left(0.31-0.31RH\right)\left(DB-14.4\right)]$ [34]. Here, $T_{max}$ and $T_{min}$ are the maximum and minimum air temperatures (°C), $T_{avg}$ is the average air temperature (°C), $RH_{Avg}$ is the average relative humidity (%), and DB and WB are dry and wet bulb temperatures (°C).

THI values from four equations were associated with test-day egg production. In the repeatability test-day model, THI was tested at every integer threshold point between 70 (THI70) and 80 (THI80) of THI. The best-fitting model was selected based on the highest coefficient of determination (R^2^) and lowest mean squared error (MSE) to determine the optimal THI threshold affecting test-day egg production characteristics.

The climate in Khon Kaen Province (northeastern Thailand) from January 2023 to July 2025 was generally hot and humid. The annual average air temperature was 27.6 °C, with the highest values recorded in April (summer) and the lowest in January (winter). The annual average relative humidity was 76.9%.

### 2.2. Genetic Estimation

The repeatability test-day model, stratified by chicken line, was used to analyze the threshold point of heat stress, decline in test-day egg production with increasing THI, estimated variance components, and genetic parameters (heritability, genetic correlations, and permanent environmental correlations) using the Average Information-Restricted Maximum Likelihood (AI-REML) algorithm [35]. The model was specified as follows:

$$y_{ijklm}={HG}_{ij}+{MIE}_{k}+{AFE}_{l}+\;\alpha \left(THI\right)+a_{0m}+\;a_{1m}\left[f\left(THI\right)\right]+\;p_{0m}+\;p_{1m}\left[f\left(THI\right)\right]+e_{ijklm}$$

where $y_{ijklm}$ is the observed value of monthly test-day egg production in hatch and generation (HG) class *ij*, months in egg (MIE) class *k*, age at first egg (AFE) covariate *l* of animal *m*; ${HG}_{ij}$ is the fixed effect of HG; ${MIE}_{k}$ is the fixed effect of MIE; ${AFE}_{l}$ is the covariate effect of AFE; $\alpha \left(THI\right)$ is the rate of decline in monthly test-day egg production per unit increase in THI); $a_{0m}$ and $p_{0m}$ are random additive genetic and permanent environmental effects without considering heat stress; $a_{1m}\left[f\left(THI\right)\right]$ and $p_{1m}\left[f\left(THI\right)\right]$ are the random additive genetic and permanent environmental effects under heat stress conditions; and $e_{ijklm}$ is the residual error. The heat stress function was defined as follows [12]:

$$f\left(THI\right)=\left\{\begin{array}{cc} 0 & ;THI\leq {THI}_{threshold}\;(no\;heat\;stress)\\ THI-{THI}_{threshold} & ;THI>{THI}_{threshold}\;(heat\;stress) \end{array}\right.$$

Heritability ($h^{2}$) was calculated as follows:

$$h^{2}=\frac{\sigma_{a0}^{2}+\sigma_{a1}^{2}+{2\sigma }_{a01}}{\sigma_{a0}^{2}+\sigma_{a1}^{2}+{2\sigma }_{a01}+\sigma_{p0}^{2}+\sigma_{p1}^{2}+{2\sigma }_{p01}{+\sigma }_{e}^{2}}$$

Genetic correlations $\left(r_{g}\right)$ and permanent environmental effect correlations $\left(r_{p}\right)$ between test-day egg production and heat stress effects were calculated as follows:

$$r_{g}=\frac{{COV\sigma }_{a0,a1}}{\sqrt{\sigma_{a0}^{2}\times \sigma_{a1}^{2}}},\;\;\;\;\;\;\;\;\;r_{p}=\frac{{COV\sigma }_{p0,p1}}{\sqrt{\sigma_{p0}^{2}\times \sigma_{p1}^{2}}}$$

## 3. Results

### 3.1. Descriptive Statistics and Identification of THI Model and Heat Stress Onset

Table 1 summarizes the data structure used to estimate variance components and genetic parameters for test-day egg production across four Thai native synthetic chicken lines. Mean test-day egg production ranged from 14.44 ± 2.5 eggs/month/bird in Soi Nin to 16.25 ± 4.4 eggs/month/bird in Kaimook e-san. Accordingly, annual egg production was the highest in Kaimook e-san (195 ± 6.2 eggs/year/bird) and the lowest in Soi Nin (173 ± 3.7 eggs/year/bird). The mean age at first egg varied by line, with Kaen Thong showing the latest onset of lay (196 ± 26 days) and Soi Nin the earliest (175 ± 24 days). During the study period, the average air temperature was 27.6 ± 2.3 °C and the average relative humidity was 76.9 ± 8.8%, indicating a tropical climate with consistent heat stress conditions.

The regression analysis between THI and test-day egg production in the four Thai native synthetic chicken lines is summarized in Table 2. Across all THI equations (evaluated at THI levels 70–80), the R^2^ values were generally low to moderate, indicating that THI accounted for only a limited proportion of the variation in test-day egg production. However, distinct differences among equations and chicken lines were evident. For THI1, R^2^ values were the highest at 0.332–0.342 in Soi Nin, 0.343–0.352 in Soi Pet, 0.365–0.373 in Kaen Thong, and 0.368–0.375 in Kaimook e-san (hereafter, values are presented in the order: Soi Nin, Soi Pet, Kaen Thong, and Kaimook e-san). The corresponding MSE values were relatively consistent within lines, at 8.421–8.434, 8.416–8.429, 8.376–8.389, and 8.352–8.365. In contrast, THI2 showed lower R^2^ values (0.228–0.235, 0.224–0.230, 0.240–0.245, and 0.262–0.267), with MSE values varying only minimally (8.445–8.454, 8.434–8.445, 8.410–8.423, and 8.395–8.408). THI3 produced intermediate R^2^ ranges (0.281–0.290, 0.281–0.288, 0.292–0.300, and 0.315–0.321), with consistent MSE values (8.438–8.451, 8.422–8.435, 8.392–8.405, and 8.388–8.401). Finally, THI4 yielded slightly higher R^2^ values than THI3 (0.313–0.323, 0.310–0.318, 0.337–0.344, and 0.349–0.358), while the corresponding MSE values remained close to those of THI3 (8.432–8.445, 8.420–8.434, 8.381–8.395, and 8.375–8.388).

Comparative analysis revealed that THI1 consistently produced the highest R^2^ values across all lines, particularly in Kaimook e-san (up to 0.375) and Kaen Thong (up to 0.373), indicating its strongest predictive ability for test-day egg production under heat stress. Conversely, THI2 yielded the lowest R^2^ values across all lines, reflecting weaker explanatory power. Among the intermediate equations, THI4 slightly outperformed THI3, especially in Kaen Thong and Kaimook e-san, where R^2^ values nearly matched those of THI1. MSE values remained similar across equations, suggesting that differences in R^2^ were due to model fit rather than large disparities in prediction error.

### 3.2. Heritability, Genetic Correlation, and Permanent Environmental Correlation

**Figure 1:**
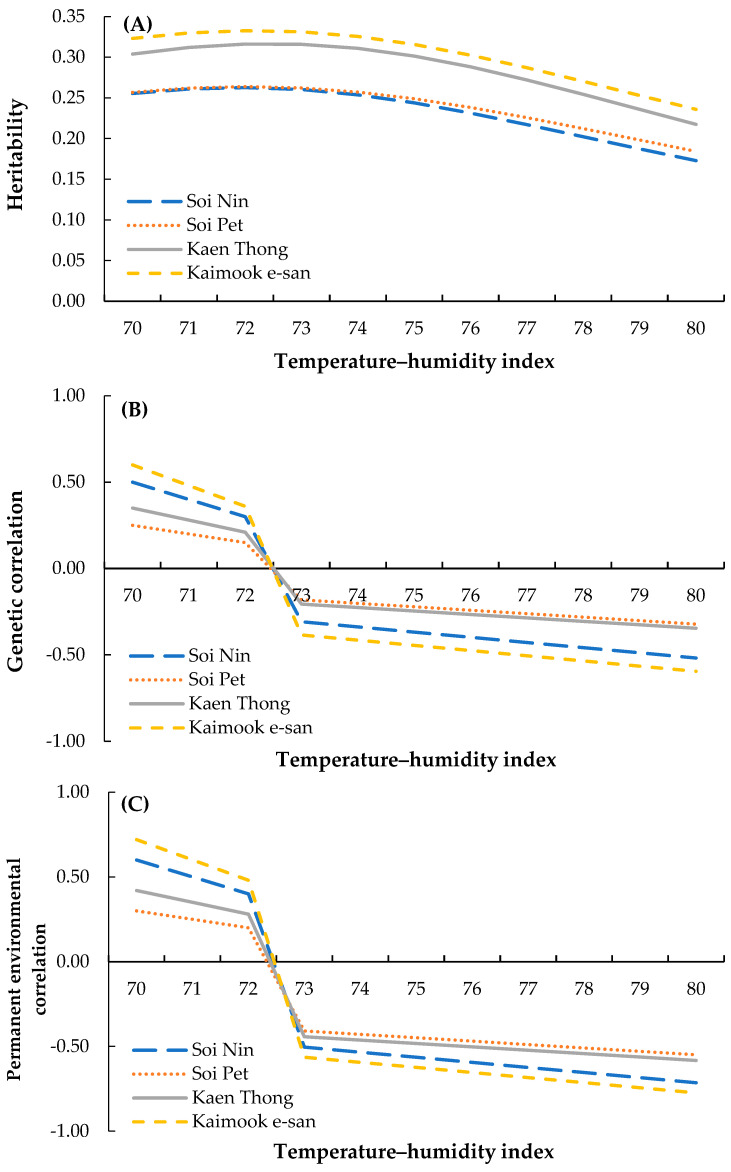
(**A**) Heritability estimates of egg production, (**B**) genetic correlations between egg production and heat stress effects, and (**C**) permanent environmental correlations between egg production and heat stress effects in four Thai native synthetic chicken lines—Soi Nin (blue), Soi Pet (orange), Kaen Thong (gray), and Kaimook e-san (yellow)—across THI values ranging from 70 to 80, calculated using the THI1 equation.

Heritability estimates for test-day egg production in four Thai native synthetic chicken lines across THI values ranging from 70 to 80 are presented in Figure 1A. In all lines, heritability estimates declined progressively with increasing THI, indicating the negative impact of environmental heat load on the proportion of phenotypic variance attributable to additive genetic effects. At THI 70, heritability was the highest, ranging from 0.255 in Soi Nin to 0.323 in Kaimook e-san. The Kaen Thong (0.304) and Kaimook e-san (0.323) lines consistently exhibited higher heritability than Soi Nin (0.255) and Soi Pet (0.257) under mild heat stress conditions. At THI 75, heritability decreased to 0.244, 0.249, 0.301, and 0.316 in Soi Nin, Soi Pet, Kaen Thong, and Kaimook e-san, respectively. Under more severe heat stress (THI 80), heritability estimates declined to their lowest levels, with values of 0.173, 0.184, 0.218, and 0.236 for Soi Nin, Soi Pet, Kaen Thong, and Kaimook e-san, respectively. The overall decline from THI 70 to THI 80 was 32.2%, 28.4%, 28.3%, and 26.9% for the Soi Nin, Soi Pet, Kaen Thong, and Kaimook e-san lines, respectively, suggesting that Kaimook e-san exhibited the greatest genetic resilience for egg production under elevated heat stress conditions.

The genetic correlations between test-day egg production and the heat stress effects are shown in Figure 1B. Across all chicken lines, positive genetic correlations were observed at lower THI values (70–72), indicating a favorable association between test-day egg production and tolerance to mild environmental conditions. At THI = 70, the correlations were 0.350, 0.250, 0.500, and 0.600 for Soi Nin, Soi Pet, Kaen Thong, and Kaimook e-san, respectively. These correlations progressively declined with increasing THI, becoming negative beyond THI = 73. By THI = 80, the correlation values reached −0.347, −0.322, −0.519, and −0.596 in the respective lines, suggesting that under severe heat stress, higher egg production was genetically associated with lower heat tolerance. Among the lines, Kaimook e-san exhibited the strongest genetic correlations (positive at low THI and negative at high THI), whereas Soi Pet consistently showed the weakest correlations.

The permanent environmental correlations between test-day egg production and heat stress effects are shown in Figure 1C. As with genetic correlations, permanent environmental correlations were positive at lower THI levels and turned negative at higher levels. At THI = 70, correlations ranged from 0.300 (Soi Pet) to 0.720 (Kaimook e-san). With increasing THI, correlations gradually declined, becoming negative beyond THI = 73. By THI = 80, correlations reached −0.583, −0.549, −0.714, and −0.774 for Soi Nin, Soi Pet, Kaen Thong, and Kaimook e-san, respectively. Kaimook e-san consistently showed the strongest absolute correlations, suggesting that its egg production was more sensitive to environmental variation under both mild and severe heat stress.

### 3.3. Rate of Decline in Traits

**Figure 2:**
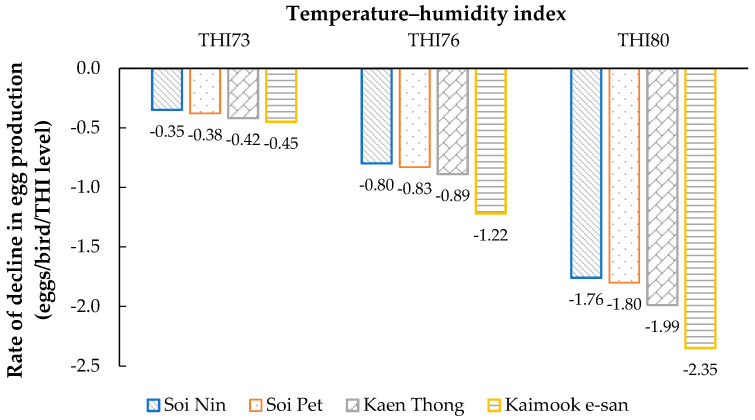
Rate of decline in egg production in four Thai native synthetic chicken lines—Soi Nin (blue), Soi Pet (orange), Kaen Thong (gray), Kaimook e-san (yellow)—at THI levels 73, 76, and 80, calculated using the THI1 equation.

The rate of decline in test-day egg production (eggs/bird/THI level) increased with rising THI in all four chicken lines (Figure 2). At mild heat stress (THI = 73), the reduction in egg production was the lowest, ranging from −0.35 eggs/bird/THI level in Soi Nin to −0.45 eggs/bird/THI level in Kaimook e-san. Under moderate heat stress (THI = 76), the rate of decline became more pronounced, with values ranging from −0.80 (Soi Nin) to −1.22 (Kaimook e-san). At severe heat stress (THI = 80), the decline was the greatest, ranging from −1.76 (Soi Nin) to −2.35 (Kaimook e-san). Across all THI levels, Kaimook e-san consistently exhibited the steepest decline, whereas Soi Nin showed the smallest decline, indicating greater tolerance to heat stress in Soi Nin compared with the other lines.

## 4. Discussion

This study addresses a key challenge for poultry in tropical regions: sustaining egg production under high temperature–humidity conditions. Estimating genetic parameters from test-day records provides insights into heritability, genetic correlations, and heat stress resilience of each line. These findings enable more accurate selection strategies, improve genetic evaluation models, and support the development of thermotolerant, productive chicken lines. Ultimately, this study contributes to enhanced food security, farmer livelihoods, and sustainable poultry breeding in hot-climate environments.

Descriptive statistics revealed notable differences in productive performance among the four Thai native synthetic chicken lines under tropical environmental conditions. Kaimook e-san showed the highest mean test-day egg production (16.25 eggs/month/bird) and annual output (195 eggs/year/bird), whereas Soi Nin exhibited the lowest values. These results are consistent with previous studies showing that genetic background significantly influences egg production potential, even within native or synthetic populations [36,37]. The superior performance of the Kaimook e-san line suggests a genetic advantage for egg-laying performance under comparable management conditions. The variation in age at first egg (AFE) among the lines—ranging from 175 days in Soi Nin to 196 days in Kaen Thong—highlights the influence of genotype on sexual maturity. Delayed AFE, as observed in Kaen Thong, can lead to higher peak production but may reduce laying persistency under environmental stress due to physiological constraints [38]. In contrast, earlier AFE, as in Soi Nin, may favor early egg output but could increase susceptibility to environmental stressors because of incomplete physiological maturity at the onset of lay [39,40]. The average ambient conditions during the study (27.6 °C and 76.9% relative humidity) indicate that hens experienced persistent thermal stress throughout the laying cycle. Such climatic conditions correspond to a THI above the typical heat stress threshold for laying hens (THI ≈ 70–72) [14,22]. Prolonged exposure to THI values above this threshold can reduce feed intake, disrupt endocrine function, and impair reproductive performance in poultry [13,41]. This is consistent with the decline in test-day egg production observed across all lines, particularly the pronounced reduction in Kaimook e-san at THI 80.

The decline in heritability estimates for test-day egg production across increasing THI values in all four chicken lines reflects the well-established negative effect of heat stress on the expression of additive genetic variance for production traits. Under thermoneutral conditions (THI ≈ 70), moderate heritability values (0.255–0.323) suggested the feasibility of genetic selection for egg production in these lines. However, as THI increased to 80, heritability declined by 26.9–32.2%, supporting previous findings that heat stress reduces the proportion of phenotypic variance attributable to additive genetic effects by increasing environmental variance and masking genetic potential [22,42]. This decline indicates that selection efficiency for egg production may be compromised under high thermal stress unless breeding programs explicitly account for genotype-by-environment interactions (GxE) [43,44,45]. Among the four lines, Kaimook e-san consistently maintained higher heritability estimates under elevated THI, indicating relatively greater resilience of genetic expression to heat stress. Similar patterns of line- or breed-specific resilience have been observed in tropical-adapted and temperate commercial layers, where local or synthetic breeds exhibited smaller declines in heritability under heat stress [46,47]. This resilience in Kaimook e-san may reflect an adaptive genetic background or selection history favoring both productivity and thermotolerance.

The genetic correlations between egg production and heat stress effects shifted from positive at low THI (0.250–0.600) to negative at high THI (−0.322 to −0.596), highlighting a potential antagonistic relationship between these traits under severe heat stress [22]. Positive correlations at lower THI suggest that genetic improvement for egg production could coincide with enhanced heat tolerance under mild conditions. Conversely, the strong negative correlations at THI 80 indicate that genotypes with higher genetic merit for egg production tended to exhibit reduced tolerance to heat stress, consistent with findings in commercial layers and broiler breeders under tropical and subtropical environments [48]. Kaimook e-san exhibited the largest magnitude of genetic correlations (both positive and negative), indicating greater sensitivity of genetic performance to environmental changes. While this may be disadvantageous under prolonged heat stress, it also suggests that targeted selection under controlled environments could yield substantial gains in productivity. Future improvements in heat tolerance may be achieved by selecting for specific heat shock protein (HSP) genes. Polymorphisms in HSP genes, such as HSP70, have been associated with improved egg production traits in various chicken breeds, suggesting a potential genetic pathway for enhancing thermotolerance [49,50].

Permanent environmental correlations exhibited a similar trend, shifting from positive at THI 70 (0.300–0.720) to negative at THI 80 (−0.549 to −0.774). This indicates that non-genetic, persistent environmental factors—such as management practices, housing, and health status—interact with climatic stressors to influence performance across the laying cycle. The stronger absolute correlations in Kaimook e-san suggest that, despite its genetic resilience, this line may be more sensitive to consistent environmental conditions across test days, which could be addressed through improved housing, ventilation, or heat-mitigation strategies [51,52]. Overall, these findings highlight the importance of incorporating both genetic and environmental sensitivity parameters into selection indices for Thai native synthetic chicken lines, especially under projected tropical climate change scenarios. Lines such as Kaimook e-san and Kaen Thong, which maintain relatively higher heritability under heat stress, represent valuable genetic resources for breeding thermotolerant, high-producing chicken lines. However, the antagonistic genetic correlations observed at high THI underscore the importance of multi-trait selection strategies that carefully balance productivity and heat resilience.

The present study showed that the rate of decline in test-day egg production increased progressively with rising THI, indicating the negative impact of heat stress on laying performance across all four lines. This aligns with previous reports linking elevated THI values to reduced egg production due to reduced feed intake, altered endocrine function, and greater energy expenditure for thermoregulation [14,53,54]. At mild heat stress (THI = 73), egg production decline was minimal, suggesting that the birds maintained performance within a narrow thermal comfort zone. However, as THI increased to 76 and 80, the decline was markedly greater, reflecting cumulative physiological strain from prolonged exposure to high temperature and humidity. Such reductions may result from disrupted ovarian follicular development, suppressed luteinizing hormone secretion, and oxidative damage to reproductive tissues, as reported in heat-stressed laying hens [16,41,55].

Among the four lines, Kaimook e-san consistently exhibited the steepest decline in egg production across all THI levels, indicating higher sensitivity to heat stress. This susceptibility may reflect genetic differences in thermotolerance, metabolic heat production, or heat dissipation capacity, which warrant further investigation. In contrast, Soi Nin exhibited the smallest decline in egg production, suggesting greater resilience under heat stress. Our findings suggest a biologically plausible trade-off between selection for high egg output and resilience to heat. Egg production is an energetically demanding process; under elevated ambient temperatures, birds selected for maximal output may have less physiological capacity to maintain thermoregulation and reproductive function. This can lead to sharper performance declines due to reduced feed intake, respiratory alkalosis, electrolyte imbalance, and impaired shell formation. Controlled trials confirm that heat stress depresses feed intake, laying rate, and egg quality, disproportionately affecting high-metabolic phenotypes [14].

From a quantitative-genetic perspective, this trade-off can be addressed by (i) modeling production as a reaction norm across THI and selecting on both level (intercept) and robustness (slope), (ii) using multi-trait selection indices that explicitly weight heat-tolerance EBVs alongside egg number, and (iii) incorporating resilience indicators derived from longitudinal egg records, which can be selected without major compromise to production. Molecular genetic approaches also offer effective alternatives. For example, polymorphisms in HSP genes and genes associated with reduced feather cover contribute to better productivity under high-temperature conditions [18,56,57,58]. An integrated breeding strategy is required to develop chickens that combine high egg production with strong heat tolerance. Genomic selection can identify birds carrying favorable alleles for both traits, while marker-assisted selection can target specific loci associated with thermotolerance. Crossbreeding high-yielding commercial layers with heat-adapted native lines can generate heterosis, and within-line selection ensures long-term improvement. In parallel, optimized management practices—such as improved nutrition, ventilation, and lighting—remain essential to maximize performance under heat stress.

## 5. Conclusions

This study evaluated test-day egg production and genetic responses to heat stress in four Thai native synthetic chicken lines under tropical conditions. Test-day egg production declined with increasing temperature and humidity, with Kaimook e-san showing the steepest reduction and Soi Nin the least. These patterns highlight genetic differences in heat tolerance, as some lines maintained performance more effectively under stress. Breeding from heat-resilient lines may help sustain productivity during hot conditions, providing valuable strategies for both smallholder and commercial poultry production in tropical regions facing rising temperatures due to climate change.

## Figures and Tables

**Table 1:** Data structure for estimation of variance components and genetic parameters in four Thai native synthetic chicken lines.

Categories	Total	Thai Native Synthetic Chicken Lines			
		Soi Nin	Soi Pet	Kaen Thong	Kaimook e-San
Animals with records (n)	1134	233	354	181	366
Animals with pedigrees (n)	2898	616	858	512	912
Number of records (n)	11,887	2330	3540	1991	4026
Average test-day egg production (eggs/month/bird ± SD)		14.44 ± 2.5	14.86 ± 3.0	15.14 ± 3.8	16.25 ± 4.4
Average annual egg production (eggs/year/bird ± SD)		173 ± 3.7	178 ± 4.0	182 ± 5.6	195 ± 6.2
Average age at first egg (days ± SD)		175 ± 24	177 ± 29	196 ± 26	185 ± 26
Average air temperature (°C ± SD)	27.6 ± 2.3	−	−	−	−
Average relative humidity (% ± SD)	76.9 ± 8.8	−	−	−	−

**Table 2:** Regression analysis of statistical parameters (R^2^ and MSE) for the relationship between temperature–humidity index (THI) and test-day egg production in four Thai native synthetic chicken lines.

THI Equations	THI Levels	Thai Native Synthetic Chicken Lines							
		Soi Nin		Soi Pet		Kaen Thong		Kaimook e-san	
		R^2^	MSE	R^2^	MSE	R^2^	MSE	R^2^	MSE
THI1	70	0.337	8.427	0.348	8.422	0.368	8.382	0.371	8.358
	71	0.338	8.425	0.349	8.420	0.370	8.380	0.373	8.356
	72	0.339	8.424	0.350	8.419	0.371	8.379	0.375	8.355
	73	0.342	8.421	0.352	8.416	0.373	8.376	0.375	8.352
	74	0.341	8.422	0.351	8.417	0.372	8.377	0.374	8.353
	75	0.340	8.423	0.350	8.418	0.371	8.378	0.373	8.354
	76	0.338	8.424	0.349	8.419	0.370	8.379	0.373	8.355
	77	0.338	8.426	0.348	8.421	0.369	8.381	0.372	8.357
	78	0.336	8.428	0.347	8.423	0.368	8.383	0.371	8.359
	79	0.333	8.431	0.345	8.426	0.366	8.386	0.369	8.362
	80	0.332	8.434	0.343	8.429	0.365	8.389	0.368	8.365
THI2	70	0.231	8.454	0.227	8.441	0.243	8.419	0.264	8.404
	71	0.232	8.451	0.228	8.438	0.244	8.416	0.265	8.401
	72	0.233	8.450	0.229	8.437	0.245	8.415	0.267	8.400
	73	0.235	8.445	0.230	8.432	0.245	8.410	0.267	8.395
	74	0.234	8.447	0.229	8.434	0.244	8.412	0.266	8.397
	75	0.234	8.448	0.228	8.435	0.244	8.413	0.266	8.398
	76	0.232	8.449	0.228	8.436	0.244	8.414	0.266	8.399
	77	0.232	8.450	0.228	8.437	0.243	8.415	0.266	8.400
	78	0.231	8.452	0.227	8.439	0.242	8.417	0.264	8.402
	79	0.229	8.455	0.225	8.442	0.241	8.420	0.263	8.405
	80	0.228	8.458	0.224	8.445	0.240	8.423	0.262	8.408
THI3	70	0.286	8.447	0.285	8.431	0.297	8.401	0.318	8.397
	71	0.287	8.444	0.286	8.428	0.299	8.398	0.319	8.394
	72	0.287	8.443	0.287	8.427	0.299	8.397	0.321	8.393
	73	0.290	8.438	0.288	8.422	0.300	8.392	0.321	8.388
	74	0.288	8.440	0.287	8.424	0.299	8.394	0.320	8.390
	75	0.288	8.441	0.286	8.425	0.299	8.395	0.320	8.391
	76	0.287	8.442	0.286	8.426	0.298	8.396	0.320	8.392
	77	0.287	8.443	0.285	8.427	0.297	8.397	0.319	8.393
	78	0.285	8.445	0.284	8.429	0.296	8.399	0.318	8.395
	79	0.283	8.448	0.282	8.432	0.294	8.402	0.316	8.398
	80	0.281	8.451	0.281	8.435	0.292	8.405	0.315	8.401
THI4	70	0.318	8.437	0.314	8.426	0.341	8.387	0.354	8.380
	71	0.319	8.436	0.315	8.425	0.342	8.386	0.356	8.379
	72	0.322	8.434	0.316	8.423	0.343	8.384	0.357	8.377
	73	0.323	8.432	0.318	8.420	0.344	8.381	0.358	8.375
	74	0.321	8.433	0.317	8.422	0.343	8.383	0.357	8.376
	75	0.321	8.435	0.316	8.424	0.342	8.385	0.356	8.378
	76	0.319	8.437	0.315	8.426	0.342	8.387	0.355	8.380
	77	0.319	8.438	0.315	8.427	0.341	8.388	0.354	8.381
	78	0.317	8.440	0.313	8.429	0.340	8.390	0.352	8.383
	79	0.315	8.442	0.312	8.431	0.339	8.392	0.351	8.385
	80	0.313	8.445	0.310	8.434	0.337	8.395	0.349	8.388

R^2^ = coefficient of determination; MSE = mean squared error.

## Data Availability

The original contributions presented in the study are included in the article; further inquiries can be directed to the corresponding author.
